# Sex Differences in Cerebrovascular Reactivity to Hypercapnia in Older Adults

**DOI:** 10.1002/alz.093323

**Published:** 2025-01-03

**Authors:** Allison C Engstrom, Arunima Kapoor, Shubir Dutt, John Paul M Alitin, Aimée Gaubert, Trevor Lohman, Isabel Sible, Anisa J Marshall, Fatemah Shenasa, Xingfeng Shao, Danny JJ Wang, Daniel A. Nation

**Affiliations:** ^1^ University of California, Irvine, Irvine, CA USA; ^2^ University of Southern California, Los Angeles, CA USA

## Abstract

**Background:**

Cerebrovascular reactivity (CVR) is a marker of cerebrovascular function defined by blood flow changes in response to vasoactive stimuli, which may represent a valuable indicator of risk for vascular brain injury and cognitive decline. Understanding of sex specific CVR patterns is limited, yet likely important for understanding vascular risk. Previous research on sex differences in CVR is mixed, and has predominantly focused on younger adults and utilized Transcranial Doppler ultrasound approaches that focus on large intracranial artery flow. We sought to determine sex differences in CVR responses in older adults using MRI approaches focused on microvascular vasodilatory responses.

**Method:**

Independently living older adults, (N = 127, mean age = 69.9 years; SD = 7.27; age range 55‐90 years, 34.6% male, mean education = 16.7 years; SD = 2.2) free of dementia, clinical stroke, and major neurological or psychiatric disorder, were recruited from the community and underwent brain MRI and clinical interview. Pseudo‐continuous arterial spin‐labeling MRI and capnography was used to quantify whole brain cerebral perfusion during CVR to hypercapnia induced by visually guided, 15sec breath‐hold exercises. Sex differences in whole brain CVR to hypercapnia were studied.

**Result:**

An independent samples t‐test revealed women (*M* = 6.52, SD = 5.40) to have significantly higher CVR to hypercapnia than men *(M* = 4.24, SD = 4.20), *t*(125) = 2.63, *p* = 0.01, which remained significant in an ANCOVA model after correcting for age *F*(125) = 4.407, *p* = 0.038.

**Conclusion:**

Older men exhibited lower cerebral vasodilatory responses to hypercapnia relative to women, independent of the effects of aging. These results are consistent with prior findings suggesting that women have higher cerebral blood flow relative to age‐matched men. Findings could suggest older women are at increased risk of vascular brain injury and resulting cognitive decline, and inform future studies of CVR as a marker of risk in this population.

